# *N*-(2-Eth­oxy­phen­yl)formamide

**DOI:** 10.1107/S160053681200205X

**Published:** 2012-01-21

**Authors:** Mohammad Kazem Rofouei, Jafar Attar Gharamaleki, Fereshteh Younesian, Giuseppe Bruno, Hadi Amiri Rudbari

**Affiliations:** a Faculty of Chemistry, Tarbiat Moallem University, Tehran, Iran; b Department of Chemistry, Islamic Azad University, Tehran Central Branch, Tehran, Iran; c Dipartimento di Chimica Inorganica, Universita di Messina, Messina, Italy

## Abstract

The title compound, C_9_H_11_NO_2_, was obtained as an unexpected product in an attempt to synthesize a triazene ligand. The title mol­ecule is almost planar, with the formamide and eth­oxy groups oriented at 2.7 (3) and 12.9 (2)°, respectively, with respect to the mean plane of the benzene ring. In the crystal, mol­ecules are linked by inter­molecular N—H⋯O hydrogen bonds, forming a chain along the *a* axis. Weak C—H⋯π inter­actions with an H⋯π distance of 2.78 Å reinforce the crystal packing, resulting in a three-dimensional network.

## Related literature

For preparation of several trizene compounds in our laboratory, see: Melardi *et al.* (2011 ▶). For similar crystal structures, see: Landman *et al.* (2011 ▶); Chitanda *et al.* (2008 ▶); Hu *et al.* (2010 ▶).
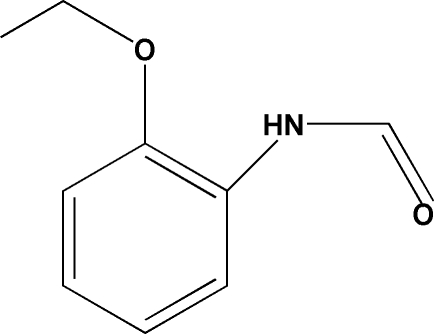


## Experimental

### 

#### Crystal data

C_9_H_11_NO_2_
*M* *_r_* = 165.19
Orthorhombic, 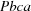

*a* = 7.9079 (4) Å
*b* = 14.1253 (6) Å
*c* = 15.9555 (7) Å
*V* = 1782.25 (14) Å^3^
*Z* = 8
Mo *K*α radiation
μ = 0.09 mm^−1^
*T* = 296 K
0.45 × 0.23 × 0.18 mm

#### Data collection

Bruker APEXII CCD diffractometer
Absorption correction: multi-scan (*SADABS*; Bruker, 2007) ▶ *T*_min_ = 0.671, *T*_max_ = 0.746
8725 measured reflections
1961 independent reflections
1248 reflections with *I* > 2σ(*I*)
*R*_int_ = 0.024

#### Refinement

*R*[*F* ^2^ > 2σ(*F* ^2^)] = 0.043
*wR*(*F* ^2^) = 0.120
*S* = 1.03
1961 reflections
110 parameters
H-atom parameters constrained
Δρ_max_ = 0.12 e Å^−3^
Δρ_min_ = −0.14 e Å^−3^

### 

Data collection: *APEX2* (Bruker, 2007 ▶); cell refinement: *SAINT* (Bruker, 2007 ▶); data reduction: *SAINT*; program(s) used to solve structure: *SHELXS97* (Sheldrick, 2008 ▶); program(s) used to refine structure: *SHELXL97* (Sheldrick, 2008 ▶); molecular graphics: XPW in *SHELXTL* (Sheldrick, 2008 ▶); software used to prepare material for publication: *SHELXTL*.

## Supplementary Material

Crystal structure: contains datablock(s) I, global. DOI: 10.1107/S160053681200205X/pv2505sup1.cif

Structure factors: contains datablock(s) I. DOI: 10.1107/S160053681200205X/pv2505Isup2.hkl

Supplementary material file. DOI: 10.1107/S160053681200205X/pv2505Isup3.cml

Additional supplementary materials:  crystallographic information; 3D view; checkCIF report

## Figures and Tables

**Table 1:** Hydrogen-bond geometry (Å, °) *Cg* is the centroid of the C3–C9 ring.

*D*—H⋯*A*	*D*—H	H⋯*A*	*D*⋯*A*	*D*—H⋯*A*
N1—H1⋯O2^i^	0.86	2.24	2.9741 (18)	144
C2—H2*A*⋯*Cg*^ii^	0.97	2.78	3.5853 (19)	141

Symmetry codes: (i) 
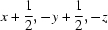
; (ii) 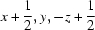.

**Fig. 1.**
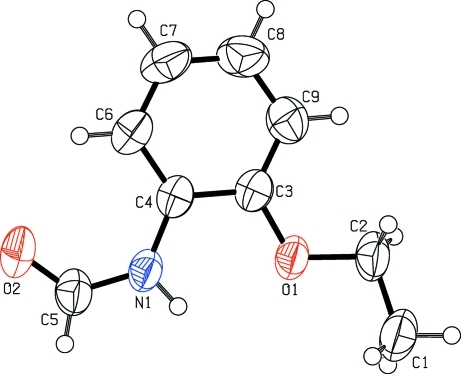
The molecular structure of the title compound with the atom numbering scheme. Displacement ellipsoids are drawn at the 50% probability level. H atoms are presented as small spheres of arbitrary radius.

**Fig. 2.**
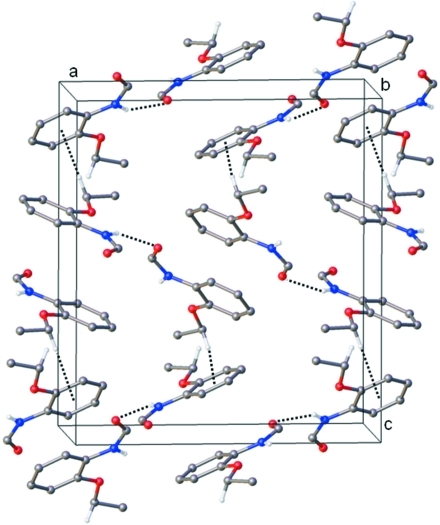
A view of the N—-H···O hydrogen bonds and and C—H···π interactions (dotted lines) in the crystal structure of the title compound. H atoms non-participating in hydrogen-bonding were omitted for clarity.

## supplementary crystallographic information

### Comment

The preparation of several trizene compounds as ligands in our laboratory has already been reported (Melardi *et al.*, 2011). However, the title compound was formed as an unexpected product in an attempt for the synthesis of a triazene ligand, 1-(2-methylphenyl)-3(2-ethoxyphenyl)triazene). In this article, we report the synthesis and crystal structure of the title compound.

The title molecule (Fig. 1) is almost planar with formamide and ethoxy groups oriented at 2.7 (3) and 12.9 (2)°, respectively, with respect to the mean-plane of the benzene ring. The bond lengths and angles in the title molecule are in accord with the corresponding bond lengths and angles reported in a few similar structures (Landman *et al.*, 2011; Chitanda *et al.*, 2008; Hu *et al*., 2010).

In the crystal structure molecules are linked by intermolecular N—H···O hydrogen bonds (Table 1) to form a chain along the *a*-axis. Weak edge-to-face C—H···*Cg*1 stacking interaction between an ethoxy hydrogen and a benzene ring with H···π distance of 2.78 Å, (*Cg*1 is the center of benzene ring atoms C3/C4/C6—C9) reinforce the crystal packing resulting in a three-dimensional network (Fig. 2).

### Experimental

The title compound was obtained as an unexpected product in an attempt for the synthesis of a triazene ligand, 1-(2-methylphenyl)-3(2-ethoxyphenyl)triazene). A 100 ml flask was charged with 10 g of ice and 15 ml of water and then cooled to 273 K in an ice-bath. To this were added 2-methylaniline (0.215 g, 2 mmol), hydrochloric acid (36.5%, 2 mmol) and 2 ml water. To this solution was then added a solution containing NaNO_2_ (0.16 g, 2 mmol) in 2 ml water during a 15 min period. After mixing for 15 min, the obtained solution was added to a solution of o-phenetidin (0.261 ml, 2 mmol), 2 ml methanol and 2 ml water. After that a solution containing sodium acetate (2.95 g, 36 mmol) in 10 ml water was added. After mixing for 24 h the colorless material was filtered off and dissolved in DMSO. By recrystallization from DMSO, the crystals of the title compound were obtained instead of the expected triazene.

### Refinement

The H atoms were placed in calculated positions and refined as riding, with N—H = 0.86 Å and C—H = 0.93, 0.96 and 0.97 Å for aryl, methy and methylene type H-atoms, respectively, with *U*_iso_(H) = 1.2–1.5 *U*_eq_(C/N).

### Crystal data

**Table:**

C_9_H_11_NO_2_	*F*(000) = 704
*M**_r_* = 165.19	*D*_x_ = 1.231 Mg m^−^^3^
Orthorhombic, *P**b**c**a*	Mo *K*α radiation, λ = 0.71073 Å
Hall symbol: -P 2ac 2ab	Cell parameters from 2150 reflections
*a* = 7.9079 (4) Å	θ = 2.6–23.1°
*b* = 14.1253 (6) Å	µ = 0.09 mm^−^^1^
*c* = 15.9555 (7) Å	*T* = 296 K
*V* = 1782.25 (14) Å^3^	Cubic, colourless
*Z* = 8	0.45 × 0.23 × 0.18 mm

### Data collection

**Table:**

Bruker APEXII CCD diffractometer	1961 independent reflections
Radiation source: fine-focus sealed tube	1248 reflections with *I* > 2σ(*I*)
graphite	*R*_int_ = 0.024
φ and ω scans	θ_max_ = 27.1°, θ_min_ = 3.2°
Absorption correction: multi-scan (*SADABS*; Bruker, 2007)	*h* = −10→10
*T*_min_ = 0.671, *T*_max_ = 0.746	*k* = −18→17
8725 measured reflections	*l* = −20→15

### Refinement

**Table:**

Refinement on *F*^2^	Primary atom site location: structure-invariant direct methods
Least-squares matrix: full	Secondary atom site location: difference Fourier map
*R*[*F*^2^ > 2σ(*F*^2^)] = 0.043	Hydrogen site location: inferred from neighbouring sites
*wR*(*F*^2^) = 0.120	H-atom parameters constrained
*S* = 1.03	*w* = 1/[σ^2^(*F*_o_^2^) + (0.0514*P*)^2^ + 0.278*P*] where *P* = (*F*_o_^2^ + 2*F*_c_^2^)/3
1961 reflections	(Δ/σ)_max_ < 0.001
110 parameters	Δρ_max_ = 0.12 e Å^−^^3^
0 restraints	Δρ_min_ = −0.14 e Å^−^^3^

### Special details

**Table:**

Geometry. All s.u.'s (except the s.u. in the dihedral angle between two l.s. planes) are estimated using the full covariance matrix. The cell s.u.'s are taken into account individually in the estimation of s.u.'s in distances, angles and torsion angles; correlations between s.u.'s in cell parameters are only used when they are defined by crystal symmetry. An approximate (isotropic) treatment of cell s.u.'s is used for estimating s.u.'s involving l.s. planes.
Refinement. Refinement of *F*^2^ against ALL reflections. The weighted *R*-factor *wR* and goodness of fit *S* are based on *F*^2^, conventional *R*-factors *R* are based on *F*, with *F* set to zero for negative *F*^2^. The threshold expression of *F*^2^ > 2σ(*F*^2^) is used only for calculating *R*-factors(gt) *etc*. and is not relevant to the choice of reflections for refinement. *R*-factors based on *F*^2^ are statistically about twice as large as those based on *F*, and *R*- factors based on ALL data will be even larger.

### Fractional atomic coordinates and isotropic or equivalent isotropic displacement parameters (Å^2^)

**Table:**

	*x*	*y*	*z*	*U*_iso_*/*U*_eq_	
O1	0.14029 (12)	0.40133 (8)	0.12756 (7)	0.0571 (3)	
N1	−0.12270 (15)	0.33831 (10)	0.04588 (8)	0.0525 (4)	
H1	−0.0300	0.3086	0.0561	0.063*	
O2	−0.37570 (15)	0.31796 (10)	−0.02102 (9)	0.0808 (4)	
C1	0.3874 (2)	0.33886 (16)	0.18902 (13)	0.0842 (7)	
H1A	0.3183	0.2875	0.2085	0.126*	
H1B	0.4759	0.3507	0.2288	0.126*	
H1C	0.4361	0.3226	0.1358	0.126*	
C2	0.2815 (2)	0.42535 (14)	0.17963 (11)	0.0653 (5)	
H2A	0.2425	0.4468	0.2340	0.078*	
H2B	0.3469	0.4758	0.1540	0.078*	
C3	0.00875 (19)	0.46374 (11)	0.12280 (10)	0.0489 (4)	
C4	−0.13409 (18)	0.43045 (11)	0.07964 (9)	0.0466 (4)	
C5	−0.23581 (19)	0.29108 (14)	0.00042 (10)	0.0597 (5)	
H5	−0.2045	0.2307	−0.0170	0.072*	
C6	−0.2759 (2)	0.48701 (12)	0.07355 (11)	0.0585 (5)	
H6	−0.3704	0.4656	0.0446	0.070*	
C7	−0.2773 (2)	0.57530 (14)	0.11042 (14)	0.0732 (6)	
H7	−0.3740	0.6126	0.1075	0.088*	
C8	−0.1370 (3)	0.60824 (13)	0.15134 (14)	0.0784 (6)	
H8	−0.1384	0.6683	0.1751	0.094*	
C9	0.0067 (2)	0.55295 (13)	0.15765 (12)	0.0661 (5)	
H9	0.1017	0.5759	0.1853	0.079*	

### Atomic displacement parameters (Å^2^)

**Table:**

	*U*^11^	*U*^22^	*U*^33^	*U*^12^	*U*^13^	*U*^23^
O1	0.0371 (6)	0.0752 (8)	0.0591 (7)	0.0031 (5)	−0.0080 (5)	−0.0113 (5)
N1	0.0351 (6)	0.0705 (9)	0.0520 (8)	0.0038 (6)	−0.0041 (6)	−0.0075 (7)
O2	0.0456 (7)	0.0982 (10)	0.0987 (10)	0.0009 (7)	−0.0234 (6)	−0.0143 (8)
C1	0.0558 (11)	0.1186 (17)	0.0781 (13)	0.0172 (11)	−0.0227 (10)	−0.0244 (12)
C2	0.0391 (8)	0.0894 (13)	0.0675 (11)	−0.0060 (9)	−0.0073 (8)	−0.0142 (10)
C3	0.0410 (8)	0.0582 (9)	0.0476 (9)	−0.0031 (7)	0.0054 (7)	0.0057 (7)
C4	0.0390 (7)	0.0587 (10)	0.0421 (8)	−0.0028 (7)	0.0041 (6)	0.0076 (7)
C5	0.0450 (9)	0.0784 (12)	0.0556 (10)	−0.0038 (9)	−0.0016 (8)	−0.0094 (8)
C6	0.0457 (9)	0.0660 (11)	0.0638 (10)	0.0035 (8)	−0.0006 (8)	0.0135 (9)
C7	0.0614 (12)	0.0597 (11)	0.0984 (15)	0.0127 (10)	0.0058 (11)	0.0182 (10)
C8	0.0793 (14)	0.0480 (11)	0.1079 (17)	−0.0010 (10)	0.0074 (12)	0.0047 (10)
C9	0.0584 (11)	0.0632 (11)	0.0768 (13)	−0.0131 (9)	0.0000 (9)	0.0013 (9)

### Geometric parameters (Å, °)

**Table:**

O1—C3	1.3656 (18)	C3—C9	1.377 (2)
O1—C2	1.4328 (18)	C3—C4	1.404 (2)
N1—C5	1.331 (2)	C4—C6	1.380 (2)
N1—C4	1.411 (2)	C5—H5	0.9300
N1—H1	0.8600	C6—C7	1.379 (3)
O2—C5	1.2185 (19)	C6—H6	0.9300
C1—C2	1.488 (3)	C7—C8	1.369 (3)
C1—H1A	0.9600	C7—H7	0.9300
C1—H1B	0.9600	C8—C9	1.383 (3)
C1—H1C	0.9600	C8—H8	0.9300
C2—H2A	0.9700	C9—H9	0.9300
C2—H2B	0.9700		
			
C3—O1—C2	118.24 (12)	C6—C4—C3	119.61 (15)
C5—N1—C4	128.85 (14)	C6—C4—N1	123.97 (14)
C5—N1—H1	115.6	C3—C4—N1	116.40 (13)
C4—N1—H1	115.6	O2—C5—N1	127.37 (18)
C2—C1—H1A	109.5	O2—C5—H5	116.3
C2—C1—H1B	109.5	N1—C5—H5	116.3
H1A—C1—H1B	109.5	C7—C6—C4	120.02 (17)
C2—C1—H1C	109.5	C7—C6—H6	120.0
H1A—C1—H1C	109.5	C4—C6—H6	120.0
H1B—C1—H1C	109.5	C8—C7—C6	120.28 (18)
O1—C2—C1	107.60 (14)	C8—C7—H7	119.9
O1—C2—H2A	110.2	C6—C7—H7	119.9
C1—C2—H2A	110.2	C7—C8—C9	120.60 (18)
O1—C2—H2B	110.2	C7—C8—H8	119.7
C1—C2—H2B	110.2	C9—C8—H8	119.7
H2A—C2—H2B	108.5	C3—C9—C8	119.78 (17)
O1—C3—C9	125.23 (15)	C3—C9—H9	120.1
O1—C3—C4	115.08 (14)	C8—C9—H9	120.1
C9—C3—C4	119.68 (15)		
			
C3—O1—C2—C1	167.77 (15)	C4—N1—C5—O2	−0.8 (3)
C2—O1—C3—C9	6.7 (2)	C3—C4—C6—C7	−0.6 (2)
C2—O1—C3—C4	−172.08 (14)	N1—C4—C6—C7	178.25 (15)
O1—C3—C4—C6	178.00 (13)	C4—C6—C7—C8	1.7 (3)
C9—C3—C4—C6	−0.8 (2)	C6—C7—C8—C9	−1.2 (3)
O1—C3—C4—N1	−0.97 (19)	O1—C3—C9—C8	−177.42 (16)
C9—C3—C4—N1	−179.82 (14)	C4—C3—C9—C8	1.3 (3)
C5—N1—C4—C6	3.4 (3)	C7—C8—C9—C3	−0.3 (3)
C5—N1—C4—C3	−177.67 (15)		

### Hydrogen-bond geometry (Å, °)

**Table:**

Cg is the centroid of the C3–C9 ring.

**Table:**

*D*—H···*A*	*D*—H	H···*A*	*D*···*A*	*D*—H···*A*
N1—H1···O1	0.86	2.20	2.6108 (16)	109.
N1—H1···O2^i^	0.86	2.24	2.9741 (18)	144.
C2—H2A···Cg^ii^	0.97	2.78	3.5853 (19)	141

Symmetry codes: (i) *x*+1/2, −*y*+1/2, −*z*; (ii) *x*+1/2, *y*, −*z*+1/2.
